# Advanced Implantable Biomedical Devices Enabled by Triboelectric Nanogenerators

**DOI:** 10.3390/nano12081366

**Published:** 2022-04-15

**Authors:** Chan Wang, Qiongfeng Shi, Chengkuo Lee

**Affiliations:** 1 Department of Electrical and Computer Engineering, National University of Singapore, 4 Engineering Drive 3, Singapore 117576, Singapore; chanwang@nus.edu.sg (C.W.); eleshiq@nus.edu.sg (Q.S.); 2 Center for Intelligent Sensors and MEMS, National University of Singapore, 5 Engineering Drive 1, Singapore 117608, Singapore; 3 NUS Suzhou Research Institute (NUSRI), Suzhou Industrial Park, Suzhou 215123, China; 4 NUS Graduate School-Integrative Sciences and Engineering Program (ISEP), National University of Singapore, Singapore 119077, Singapore

**Keywords:** implantable biomedical devices, triboelectric nanogenerator, energy harvester, self-powered biosensor, nerve stimulation, muscle stimulation

## Abstract

Implantable biomedical devices (IMDs) play essential roles in healthcare. Subject to the limited battery life, IMDs cannot achieve long-term in situ monitoring, diagnosis, and treatment. The proposal and rapid development of triboelectric nanogenerators free IMDs from the shackles of batteries and spawn a self-powered healthcare system. This review aims to overview the development of IMDs based on triboelectric nanogenerators, divided into self-powered biosensors, in vivo energy harvesting devices, and direct electrical stimulation therapy devices. Meanwhile, future challenges and opportunities are discussed according to the development requirements of current-level self-powered IMDs to enhance output performance, develop advanced triboelectric nanogenerators with multifunctional materials, and self-driven close-looped diagnosis and treatment systems.

## 1. Introduction

Implantable biomedical devices (IMDs) have received rapid development in recent decades, effectively improving patients’ life quality and prolonging lifespan [1,2,3]. Numerous IMDs have been developed in medical monitoring and treatment tools such as pacemakers [4,5], in situ blood pressure sensors [6], cardio-aid defibrillators, cochlear prosthesis, and deep brain electrical stimulators, as well as that of peripheral nerve and muscles, etc. [7,8,9,10,11,12]. The IMDs have contributed significantly to deepening the reorganization of the human body’s biological processes, including the complex mechanisms of neural communication and the formation of memory [13,14,15]. Since the birth of the first implanted pacemaker in 1958, the fabrication and application of IMDs have made significant progress but still face many challenges [16,17,18]. On the one hand, IMDs need to be more miniaturized and lightweight to weaken the impact on daily life and improve comfort [19,20]. On the other hand, the battery life is typically 3 to 5 years, and then it must be surgically removed to replace the battery, which further increases the risk and cost of patients [21,22].

Harvesting energy from human activities and physiological environments to power the operation of IMDs provides a feasible solution to miniaturize implantable biomedical systems [23,24,25,26,27,28,29,30,31,32,33]. Researchers have demonstrated that various bioenergy, from a temperature gradient, glucose oxidation, muscle contraction, et al., can be harvested and converted into electricity [34,35,36,37,38,39,40,41,42,43,44,45,46]. Among all bioenergy sources, biomechanical energy from human movements, including blood circulation, and the contraction and relaxation of the heart/lung, is considered the most abundant and effective [47]. 

Numerous efforts have been devoted to pushing forward the development of biomechanical energy harvesting technologies, mainly including piezoelectric nanogenerator (PENG) and triboelectric nanogenerator (TENG) [26,28,30,40,41,42,44,48]. Researchers have reported a piezoelectric ZnO nanowire that successfully converted the periodic vibrational energy from respiration and heartbeat into electricity, relying on the mechanical-electrical coupling effect [49]. The first in vivo biomechanical energy harvester using a TENG was proposed in 2014, which outputted the power density of 8.44 mW/m^2^ from a rat’s normal respiration. Among them, the TENGs stand out as the biomechanical energy harvesters with the most potential for better biological compliance, high energy conversion efficiency, larger material sources and easier fabrication [50,51]. 

The triboelectric generator was first proposed by Zhong Lin Wang in 2012 [52], based on the principles of electrostatic induction and charge coupling, and was carried forward in the field of energy harvesting [53,54,55,56,57,58,59,60], wearable electronic devices [61,62,63,64,65,66,67,68,69], the Internet of Things [70,71,72,73,74,75,76,77,78,79,80], human-computer interaction [71,81,82,83,84] and security monitoring [71,85,86,87,88]. For instance, a self-powered and self-functional sock enabled by TENGs exhibited outstanding energy harvesting efficiency and multiple physiological signals monitoring performance (including gait, contact force, sweat level, et al.) [89]. The triboelectric self-powered wearable, flexible patch made applications as the 3D motion control interface in the robotic manipulator [90]. Notably, a series of implantable TENGs which demonstrated its potential for in vivo physiological signal sensors and biomechanical energy harvesting from heart rate, blood pressure, and respiration were developed [91,92,93,94,95,96,97,98,99]. The biomimetic membrane sensors (BMS) based on TENGs could identify the high-frequency vocal vibrations and the low-frequency fluctuations from human pulse [100,101]. 

**Figure 1:**
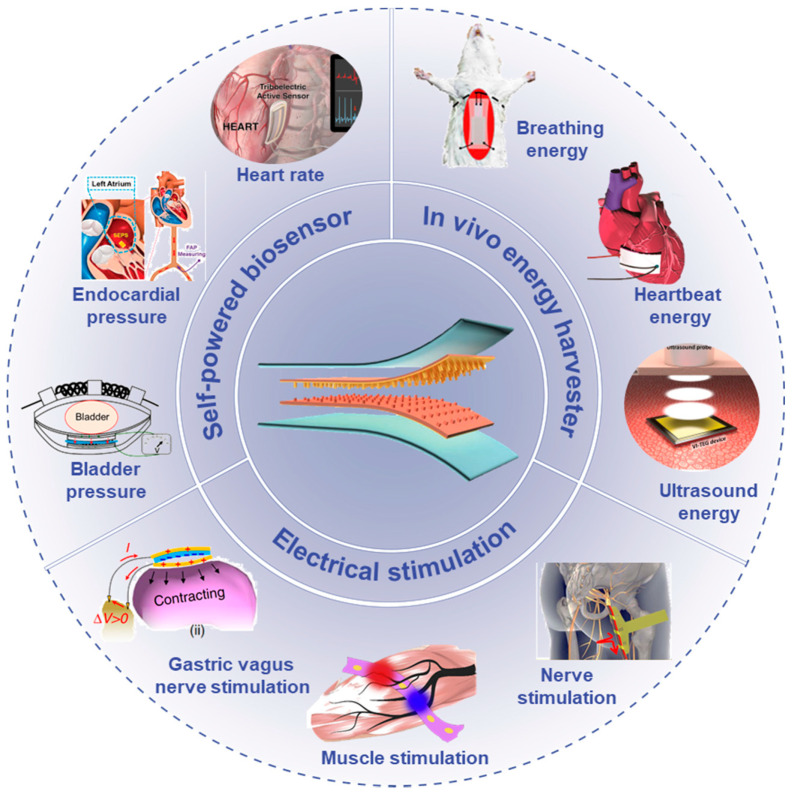
Overview of the implantable biomedical devices based on triboelectric nanogenerators (TENGs) incorporating self-powered biosensors, in vivo energy harvesters, and electrical stimulation. Reprinted with permission from Ref. [102]. Copyright 2016 American Chemical Society; Reprinted with permission from Ref. [103]. Copyright 2018 John Wiley & Sons; Reprinted with permission from Ref. [104]. Copyright 2018 American Chemical Society; Reprinted with permission from Ref. [105]. Copyright 2018 American Chemical Society; reprinted with permission from Ref. [3]. Copyright 2016 American Chemical Society; reprinted with permission from Ref. [106]. Copyright 2019 American Association for the Advancement of Science; reprinted with permission from Ref. [107]. Copyright 2017 Elsevier.; Reprinted with permission from Ref. [108]. Copyright 2019 Published by Elsevier.; Reprinted from Ref. [109]. Copyright 2018 Springer Nature.

In this review, we introduced the research progress of implantable medical devices enabled by advanced triboelectric nanogenerators incorporating self-powered biosensors, in vivo energy harvesters, and electrical stimulation, as shown in Figure 1. In Section 2, we introduced the TENG’s working principle and four working modes. Section 3 summarized the self-powered biosensors and their applications in the real-time monitoring of blood pressure, heart rhythm, and bladder pressure. Section 4 reviewed the biomechanical energy harvesters and in vivo ultrasound-driven energy harvesting devices based on TENGs. Section 5 introduced the research about directly electrical stimulators with TENGs as current sources, including electrical nerve and muscle stimulation. Section 6 summarized the advanced TENGs with biodegradable and self-healing properties. In the last section, we talked about the challenges and opportunities of the self-powered IMDs mainly from three aspects: improving output performance, developing advanced materials with multifunction, and a self-powered close-looped IMDs system.

## 2. Triboelectric Nanogenerators

**Figure 2:**
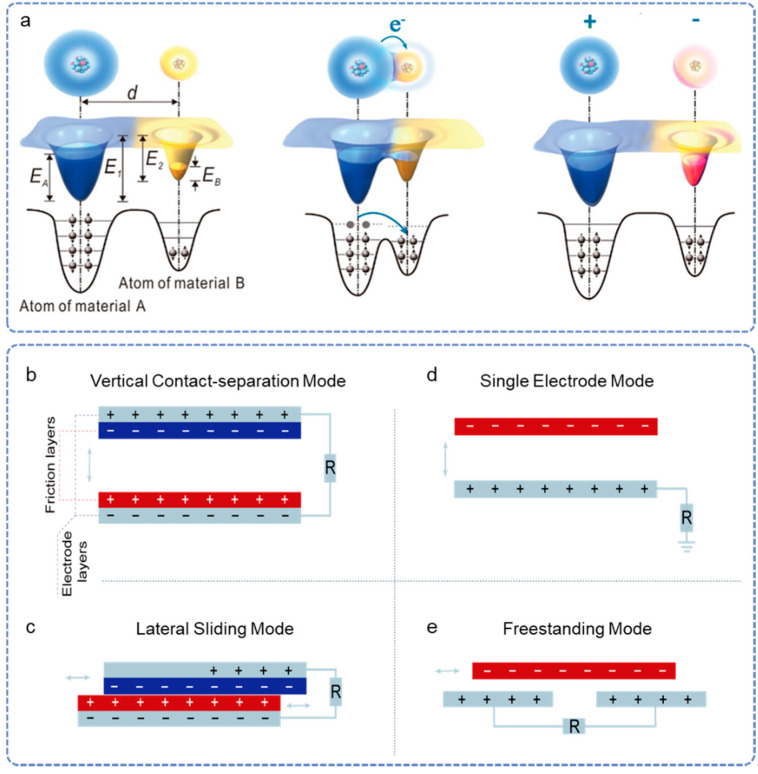
The working principle of triboelectric nanogenerators (TENGs). (**a**) Contact-electrification mechanism explained by electron cloud potential well model. Reprinted with permission from Reference [118]. Copyright 2018 John Wiley & Sons. Four modes of TENGs, including (**b**) vertical contact-separation mode, (**c**) single electrode mode, (**d**) lateral sliding mode, and (**e**) freestanding mode.

Triboelectric nanogenerators are based on triboelectric and electrostatic coupling effects [110,111,112,113,114,115,116,117]. In general, contact electrification occurs between two materials in different polarities. As shown in Figure 2a, during the contact process, the electrons transferred from the atom of material A to that of material B with their electron cloud are forced to overlap [118]. After separation, these two materials are equipped with equal positive charges and negative charges, respectively. At the same time, induced charges are generated on the electrodes under the electrostatic force from the triboelectric charges [119]. When the relative position of the two friction layers changes under the external force, the induced charges flow out and into the electrodes according to the corresponding change in the electric field [120,121,122,123]. By connecting with an electrical loading between two electrodes, the electrical pulses could be recorded, related to the change rate of induced charges, and satisfy the equation of Maxwell’s displacement current [124].

$$J_{D}=\frac{\partial D}{\partial t}=\varepsilon \frac{\partial E}{\partial t}+\frac{\partial P_{s}}{\partial t}$$

where *J_D_* is the density of free conduction current density in space due to charge flow, *D* is the electric displacement vector, *t* is time, *E* is the electric field, *P_s_* is the polarization created by the electrostatic surface charges. 

Triboelectrification or contact electrification exists widely in our lives, enabling an extensive selection of friction materials [125,126,127,128]. The structure of TENG could be designed according to the application scenario requirements [70,71,129,130,131,132,133]. As shown in Figure 2b–e, there are mainly four working modes, including a vertical contact-separation (CS) mode, single electrode (SE) mode, lateral sliding (LS) mode, and freestanding (FS) mode [124,134,135,136]. 

### 2.1. Vertical Contact-Separation Mode

The CS mode is first reported as the most basic TENG mode, which usually consists of two dielectric materials as the friction layers and the metal deposited on their back surface as electrodes (Figure 2a). Under external force, the two friction layers continuously contact and separate from each other. Meanwhile, the induced charges in the electrodes flow across the external circuit driven by the electrical field. The TENGs based on CS mode possessing the advantages of simple structure, easy preparation, and good output performance have substantial applications in wearable sensors. In particular, it has unique advantages as implantable devices attributed to easy, full-encapsulation, robustness, and high energy conversion efficiency in vivo.

### 2.2. Lateral Sliding Mode

The LS mode TENGs have the same structure as that of CS mode TENG but triggered triboelectric charges by the relative lateral sliding of the two friction layers. During periodic sliding separation and contact, the electrostatic field drives electrons to flow between two electrodes horizontally to realize a periodic alternating output. Compared to the CS mode, the TENGs based on LS mode possess higher charge transfer efficiency due to the sufficient contact of friction layers, but poor durability. In particular, when combined with the disc rotation or cylindrical rotation, the TENGs based on LS mode can realize up to several thousand volts [135,136], which shows significant potential in wind and ocean energy harvesting [134,137]. 

### 2.3. Single Electrode Mode

There are two electrodes connected by an external load of both CS and LS mode TENGs, while SE mode has only one friction layer and a single electrode, which is grounded. The proposal of the SE mode greatly broadens the application potential of TENG, for some moving objects operated directly as the second frictional layer, like the energy harvester driven by raindrops [138]. In addition, one of the disadvantages of the SE mode TENG is the relatively small output due to the reduced efficiency of charges transferred between two triboelectric materials.

### 2.4. Freestanding Mode

TENGs based on the FS mode consist of a charged moving object and two fixed triboelectric layers. The electrodes near triboelectric layers are connected with external loading. The reciprocating moving of the charged object between the two friction layers will cause a potential change between two electrodes, which drives the electrons to flow back and forth in the external circuit loop. It is considered as the most effective mode because the FS-TENGs can generate maximum power output and the highest energy conversion efficiency compared with other modes [139]. Meanwhile, the TENGs based on the noncontact FS mode exhibit the highest robustness, since the moving charged objects do not need to contact the fixed friction layers directly. The material abrasion can be greatly reduced, which significantly improves the durability of devices.

## 3. Self-Powered Biosensors

Accurate identification and long-term monitoring of the health status of various organs in the body is the most important and basic function for in vivo medical devices, which provide original support for subsequent targeted treatment plans. Organs and tissue dysfunction often accompanied by abnormal tissue morphology and respiratory action, like cardiovascular disease related to blood pressure that is too high or too low, can be judged by monitoring physiological differences. Self-powered sensors based on TENGs show high sensitivity to slight force, pressure, and mechanical deformation in thousandth or millionth magnitude, making them ideal for in vivo health monitoring [140,141]. At the same time, the cross fusion of functional materials and advanced manufacturing technology with TENGs enables active biosensors to have better accuracy, durability and biocompatibility, and flexibility [142,143].

**Figure 3:**
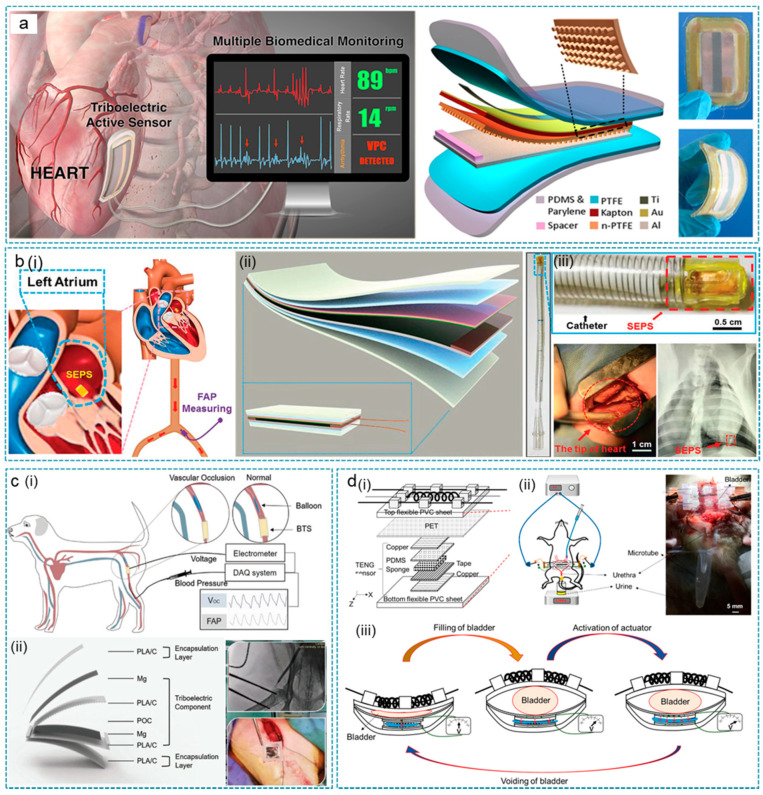
TENGs for implantable active sensors. (**a**) TENG as implantable self-powered cardiac sensors (iTES). The working principle (left) and structure (right) of the iTEAS. Reprinted with permission from Ref. [102]. Copyright 2016 American Chemical Society. (**b**) TENG as an implantable self-powered endocardial pressure sensor (SEPS). (i,iii) The SEPS implanted into an Adult Yorkshire swine’s heart. (ii) Schematic structure of the SEPS. Reprinted with permission from Ref. [103]. Copyright 2018 John Wiley & Sons. (**c**) TENG as a blood pressure sensor (BTS). The schematic diagram of BTS used for abnormal cardiac event identification (i) in a large animal and its structure (ii). Reprinted with permission from Ref. [93]. Copyright 2021 John Wiley & Sons. (**d**) TENG as a bladder pressure sensor. The schematic diagram about the structure (i) and working principle (iii) of the bladder pressure sensor. (ii) The bladder pressure sensor in vivo. Reprinted with permission from Ref. [104]. Copyright 2018 American Chemical Society.

For instance, Ma et al. proposed a multifunctional biomedical sensor (iTEAS) for providing real-time, accurate, and continuous monitoring of multiple physiological and pathological signs (Figure 3a) [102]. The iTEAS was designed in a core-shell structure with a 50 μm surface-nanostructured polytetrafluoroethylene film service as one triboelectric layer, and the 100 μm aluminum foil as both the other triboelectric layer and electrode. When driven by the small animals’ breath and breathing, the iTEAS guarantees output of 10 V (Voc) and ~4 μA (Isc). Meanwhile, the events of atrial fibrillation and premature ventricular contractions can be accurately recognized in real-time through analyzing changes in iTEAS’s output peak, which achieved a high measurement accuracy of 99% in large animals. The iTEAS can also estimate blood pressure and calculate blood flow velocity with the help of an arterial catheter. The test two weeks after the implanting operation claims good durability and bidirectional biocompatibility of the iTEAS in vivo.

Endocardial pressure status monitoring has important clinical significance for heart failure patients with impaired cardiac function. Liu et al. reported a transcatheter self-powered endocardial pressure sensor (SEPS) integrated with a surgical catheter for minimally invasive implantation [103]. As shown in Figure 3b, the oversize of SEPS was 1 × 1.5 × 0.1 cm^3^ with nano-PTFE and Al film as frictional layers. After the surface nanostructure and corona discharge process of one frictional layer, the open-circuit voltage of SEPS was enhanced from 1.2 V to 6.2 V. In the in vitro testing system, the SEPS demonstrated outstanding linearity (R^2^ = 0.997) with a sensitivity of 1.195 mV mm/Hg at a wide pressure range from 0 to ≈350 mmHg. In an adult Yorkshire pig model, SEPS was implanted into the left ventricle for monitoring intracardiac pressure and diagnosing clinical conditions such as arrhythmias, ventricular fibrillation and premature ventricular contractions. On this basis, Ouyang et al. reported a completely bioabsorbable triboelectric sensor (BTS), which could directly convert blood pressure changes into electrical signals (Figure 3c) [93]. The BTS demonstrated excellent sensitivity (11 mV/mmHg), linearity (R^2^ = 0.993), and good durability (450,000 cycles) with an air-gap structure. In large animals (dogs), BTS successfully identified abnormal vascular occlusion events and could be completely degraded and absorbed after maintaining stable output for five days. 

Another group, Hassani et al., developed the self-control system for neurogenic underactive bladder (UAB) by integrating a TENG sensor with a bistable micro-actuator [104]. As shown in Figure 3d, the TENG sensor was designed with PET, copper, PDMS, and wet sponge, assessed layer by layer. With the 1 mm-thick wet sponge layer as an effective spacer, the TENG sensor well-identified 0 to 6.86 N force with output increased from 35.6 mV to 114 mV, covering the bladder pressure range. The TENG behaved as the sensing component in the self-control system for detecting the fullness status of the bladder for the consequent activation of the actuator. In the in vivo experiment, the constant output of the sensor when the bladder was almost fulfilled activated the actuator to give an extrusion force on bladder and sensor. Then, the voltage-gated shape bistable actuator empties 78% volume of an anesthetized rat within 20 s relay on memory alloy components integrated on biocompatible polyvinyl chloride sheets. The as-proposed self-control bistable actuator system helps improve the quality of life and reduce pain and severe complications for UAB patients.

The implantation sites of these advanced self-powered biosensors were mainly concentrated inside/outside the heart, blood vessels, bladder and other places with large mechanical energy, as shown in Table 1. Adult Yorkshire pigs are ideal implantation animals for their similar characteristics of the cardiovascular system and weight to humans, enabling the larger electrical output. Some tissues or organs with less mechanical deformation that is still significant, such as vocal cords, tendons, ligaments, etc., should also be paid attention to for their in-situ monitoring and health status analysis. This requires IMDs to put forward higher requirements in terms of output performance, packaging technology, implantation technology and signal acquisition. At present, the long-term monitoring of most implantable self-powered biosensors relies on indwelling conductive wires, which easily leads to tissue inflammation and instability of implants. Equipping a wireless transmission module into the integration with the sensing unit and dates analysis module should be taken into consideration. Furthermore, the waveform, phase, amplitude, and change rates of the sensing dates contain rich biological health information. The in-depth analysis of these sensing dates is very important, which could be potentially realized by the rapidly advancing deep learning technologies.

## 4. In Vivo Energy Harvesters

Their direct role as bioenergy harvesters to power other implantable electronic medical devices is another important branch of the implantable TENGs. Although the self-powered biosensors are free from the constraints of bulky batteries, other components, such as the dates transmission unit, still require power sources. TENGs have been developed to harvest bioenergy from various human activities. The potential power from the shoulder movements is 2.2 W, and the corresponding power from the knee joint and ankle joint movements can reach 36.4 W and 66.8 W. Other biomechanical energy, involving heartbeat (0.93 W) and respiratory muscle (0.41 W) movement during breathing, is also potentially harvestable [38]. 

### 4.1. Bioenergy Harvester

**Figure 4:**
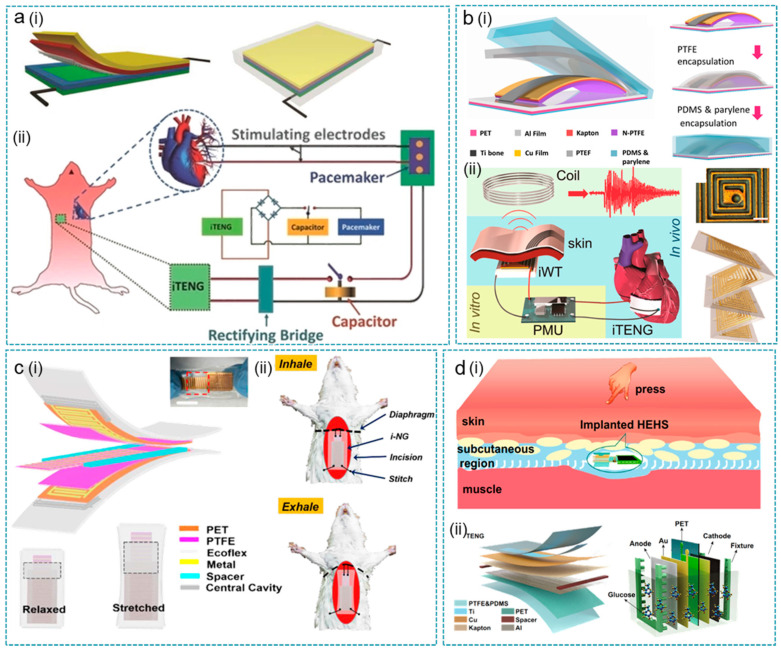
TENGs as in vivo energy harvesters. (**a**) In vivo power source for pacemaker driven by breathing. (i) The structure diagram of the implanted TENG (iTENG). (ii) Schematic diagram of iTENG for powering a pacemaker. Reprinted with permission from Ref. [144]. Copyright 2014 John Wiley & Sons. (**b**) In vivo self-powered wireless cardiac monitoring driven by heartbeat. (i) Structure diagram of the implanted TENG (iTENG). (ii) The self-powered wireless transmission system based on the iTENG. Reprinted with permission from Ref. [3]. Copyright 2016 American Chemical Society. (**c**) In vivo breath energy harvester. (i) Structure diagram of the implantable generator (i-NG). (ii) The i-NG for in vivo breath energy harvesting. Reprinted with permission from Ref. [105]. Copyright 2018 American Chemical Society. (**d**) TENG integrated with a hybrid biofuel cell (EHES) for bioenergy harvesting. (i) Conception graph of an implanted HEHS for biomechanical and biochemical energy in body. (ii) Structure diagram of the TENG and biofuel cell. Reprinted from Ref. [51]. Copyright 2020 Springer.

In recent years, TENGs for bioenergy harvesting have sprung up. In 2014, Zheng et al. first demonstrated an implanted TENG (iTENG) in a living rat to harvest bioenergy from its periodic breathing [144]. As shown in Figure 4a, the iTENG was designed to be 2 × 2 cm^2^ for the small rat thorax implantation. In one breath, the magnitude of the voltage and current signals was about 3.73 V and 0.14 µA, respectively. Then, the electrical energy was stored in a capacitor to power the pacemaker prototype. According to theoretical calculations, the energy obtained from five breaths can generate a pulse that regulates rat heart rate, which is a breakthrough in the research on self-driving implantable medical systems. The output of iTENG was further promoted by Zheng et al. by introducing a memory alloy as “keel structure”, which facilitates the contact and separation of frictional layers in complex living environments (Figure 4b) [3]. Driven by the heartbeat of an adult Yorkshire pig, the V_oc_ and I_sc_ reach 14 V and 5 μA, which are 3.5-fold and 25-fold higher than the previous one, respectively. After 72 h of implantation, the iTENG exhibited excellent stability. By connecting the iTENG within an implantable wireless transmitter, a self-powered data transmission system was fabricated for a real-time remote cardiac monitoring system.

The low frequency (<5 Hz) of human activities, such as breathing and heartbeat, leads to the discrete output of implanted TENGs, which is impractical to use directly as a power source. As shown in Figure 4c, Li et al. designed a sliding mode implantable NG (i-NG) based on grating electrodes to convert low-frequency biomechanical motions into continuous electrical signals [105]. With 200 μm of each finger in the grating-electrode, the i-NG generated seven peaks within one envelope of 0.1 s, corresponding to a frequency of 70 Hz. In an adult SD rat, the i-NG generated 7–8 voltage peaks with V_pp_ of 0.8 V (two electrode-units) during one respiration cycle. Connected with the rectifier and capacitor, i-NG could stably power a green LED without blinking. Li et al. developed a hybrid energy-harvesting system (HEHS) for both biomechanical energy and biochemical energy simultaneously collecting with the integration of an implantable triboelectric nanogenerator (TENG) and glucose fuel cell (GFC) [51]. As shown in Figure 4d, driven by pressing and hand flapping, TENG realized 22 V (V_oc_) and 0.24 μA (I_sc_), respectively. Meanwhile, GFC generated a stable output voltage of 0.6 V and 10.5 μA at three times glucose loading. After integration in parallel, HEHS obtained a superimposed current of 1.2 μA and voltage of 21.7 V, stating a higher efficiency hybrid bioenergy harvesting system. 

### 4.2. Ultrasound-Driven Energy Harvester

The above demonstrations show that directly harvesting energy from in vivo organ movements has been achieved with highly sensitive and compliant TENGs [145,146,147,148,149]. Though in vivo organs such as the heart and lung can exert regular vibrations as the internal energy sources, they generally have low frequency, low acceleration, and low amplitude that are difficult to be harvested. Especially for small animal models, the generated energy from the implantable TENGs may not be sufficient to power medical implants. On the other hand, wireless energy delivery using external sources to transfer energy to implantable energy harvesters offers an alternative approach that is more stable, adaptable, and provides power-on-demand [150]. The acoustic wave has been widely used in medical applications, including monitoring, diagnosis, and in vivo imaging. It can also penetrate deep tissue with low attenuation, and thus it is a safe and good medium for wireless energy delivery [151]. In the past few years, several implantable piezoelectric energy harvesters, as well as TENGs, have been proposed to study the performance and efficiency of acoustic energy transfer (AET) [106,132,152,153,154].

**Figure 5:**
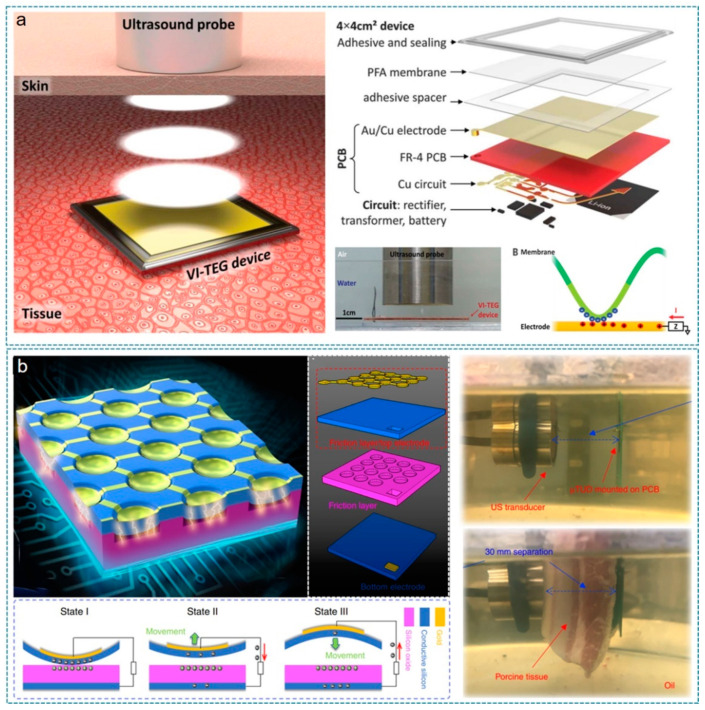
Ultrasound-driven energy harvester. (**a**) Ultrasound energy harvesting under skin based on triboelectric effects. Illustration of US energy harvesting under skin using the vibrating and implantable triboelectric generator (VI-TEG, left). The structure diagram and working principle of the VI-TEG (right). Reprinted with permission from Ref. [106]. Copyright 2019 American Association for the Advancement of Science. (**b**) Micro triboelectric ultrasonic device for acoustic energy transfer. Reprinted from Ref. [155]. Copyright 2020 Springer Nature.

In 2019, Hinchet et al. developed a vibrating and implantable triboelectric generator (VI-TEG) to harvest the wirelessly delivered ultrasound energy from a controllable external source (Figure 5a) [106]. The VI-TEG mainly consists of a thin perfluoroalkoxy (PFA) membrane (~50 µm) and an Au/Cu electrode with an airgap of 80 µm to collect the charges when the membrane vibrates under ultrasound pressure. This working mechanism triggered by the incoming ultrasound eliminates the influence of implanted site, in vivo environment, and usage scenarios. The whole device is sealed with melt adhesive and further integrated with a power management circuit and a Li-ion battery on the backside. Operated in a single-electrode mode, the VI-TEG can produce a peak output voltage of 25 V (at 40 MΩ impedance) and current of 1.3 mA (at 1 Ω impedance), when it is tested in a grounded water medium 5 mm away from a 3-cm-diameter ultrasound probe (20 kHz and 3 W/cm^2^). In an ex vivo AET experiment (20 kHz and 1 W/cm^2^) through a 0.5 cm porcine tissue, the generated outputs are more than 2.4 V and 156 µA, which are high enough as power supplies for small medical implants. 

Later in 2020, Chen et al. reported a micro-structured triboelectric ultrasonic device (μTUD) based on the technology fusion of TENG and microelectromechanical systems (MEMS) [155]. As indicated in Figure 5b, the μTUD is composed of an array of micro capacitors that has a suspended membrane over a vacuum cavity. Silicon oxide is adopted as the friction layer, while a highly conductive doped silicon layer is used as the electrode and another friction layer. When ultrasound pressure is applied to the μTUD, the suspended membranes vibrate and contact the oxide layer periodically, generating triboelectric outputs in a contact-separation mode. Ex vivo AET measurements are then performed through an oil medium (30 mm distance) without and with a sound-attenuation medium (porcine tissue). In an incident acoustic wave of 63 kPa at1 MHz, the generated output voltage reaches 16.8 mV and 12.7 mV, respectively. Even though the output power of the demonstrated μTUD is currently low, theoretical analysis by the authors shows that the device’s output power could be enhanced by 10,000 times through optimizations in material selection, geometry structure, management circuit, etc.

From direct biomechanical energy harvesting to ultrasonic-driven in vivo energy harvesting based on the triboelectric effect, IMDs have been put forward to better output performance and longer operation time, but there is still a long way to go to fully realize self-sustained and real-time IMDs. Many significant factors should be taken into consideration, and we will give a summary and discussion in Section 7.1.

## 5. Electrical Stimulation Therapy

### 5.1. Nerve Stimulation

The rapid development of flexible implantable bioelectronics has opened up a new way to enable electroceuticals to more sensitively and accurately record and modulate the bio-signals [155,156,157,158]. In this regard, neuromodulation has been widely adopted as a reversible and non-destructive therapeutic strategy to manipulate various bodily functions by stimulating or interfering with the neurophysiological signals in the neural network [159,160]. More specifically, neural signals transmitted in the nerve fibers can be tuned via stimulation on the targeted fibers for therapeutic intervention, such as restoring the sensory/motor function of neuro-prosthesis, controlling bodily functions, treating diseases, etc. [133,161,162,163,164,165,166,167,168]. Benefited by the self-generated characteristics and wearable/implantable compatibility, TENGs have been integrated with neural interfaces and explored for nerve stimulation, using the directly generated outputs from mechanical actuation [18,81,107,109,169,170,171,172].

**Figure 6:**
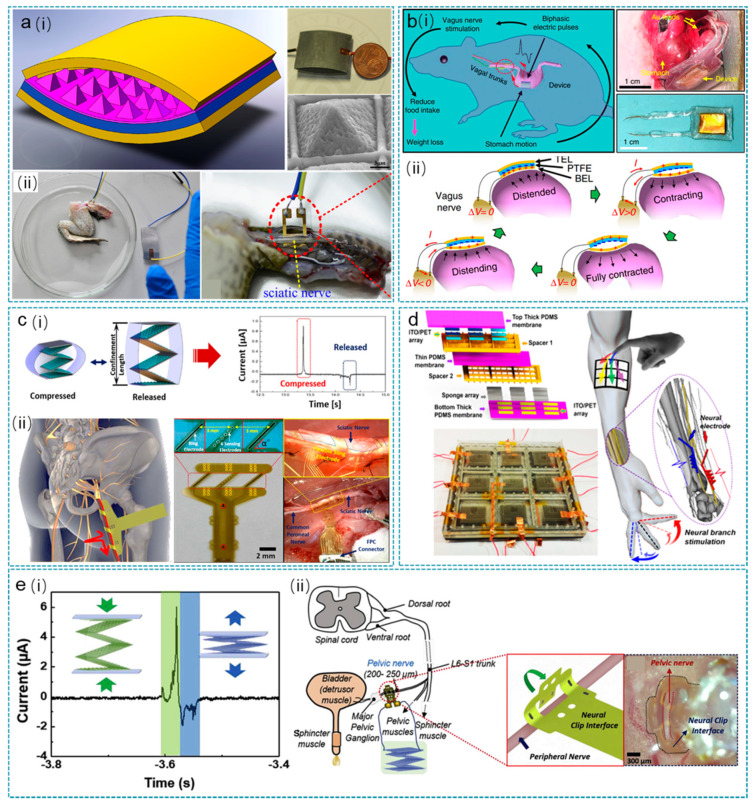
TENGs for nerve stimulation. (**a**) High-performance TENG for frog sciatic nerve stimulation. (i) The structure diagram and image of the high-performance TENG. (ii) The high performance TENG for frog sciatic nerve stimulation. Reprinted with permission from Ref. [169]. Copyright 2014 Elsevier. (**b**) Effective weight control via nerve stimulation (VNS). The schematic diagram of VNS system (i) and the working principle of VNS devices (ii). Reprinted from Ref. [109]. Copyright 2018 Springer Nature. (**c**) Modulated control of tibialis anterior muscle via common peroneal nerve stimulation based on TENGs. (i) The TENG in a compressed state and a released state, outputting voltage pulse. (ii) The TENG for tibialis anterior muscle controlling via common peroneal nerve stimulation. Reprinted with permission from Ref. [107]. Copyright 2017 Elsevier. (**d**) Peripheral nerve direct stimulation based on TENGs. Reprinted with permission from Ref. [170]. Copyright 2018 Elsevier. (**e**) Direct stimulation of autonomic pelvic nerve for restoring bladder functions. Schematically showing the working principle (i) and applications as nerve stimulation power sources (ii) of the TENG. Reprinted with permission from Ref. [171]. Copyright 2019 Elsevier.

As shown in Figure 6a, Zhang et al. first applied a high-performance TENG for a dissected frog leg [169]. The TENG is operated in the contact-separation mode, with Al and PDMS (produced with micro/nano structure and treated with fluorocarbon plasma) as the two triboelectric materials. Actuated by a 5 Hz external force, it can generate outputs of 265 V and 18.3 μA/cm^2^ in terms of voltage and current density, respectively. The outputs are then applied to the sciatic nerve of a frog leg via a 3D microneedle electrode array (MEA) toward the realization of the self-powered neural prosthesis. The two electrodes of the MEA are directly connected to the outputs from the TENG without the use of any external circuits. With force applied to the TENG, the sciatic nerve is successfully stimulated by the instantaneous outputs from the TENG, leading to the muscle actuation of the frog leg.

To obtain effective obesity treatment, Yao et al. proposed an implanted system that can respond to stomach movement and perform vagus nerve stimulation to regulate food intake (Figure 6b) [109]. The implanted system only consists of a flexible and biocompatible TENG, with its body is attached to the stomach surface while the output electrodes are directly connected to the vagus nerve. With food intake, the peristalsis motion of the stomach induces cyclic contact and separation on the TENG, which then generates biphasic electrical signals (~0.05 to 0.12 V recorded on a 1 MΩ load) to stimulate the vagal afferent fibers for food intake reduction and weight control. This strategy is then verified using rat models, and the results show that the average body weight of the stimulation group is 38% less than the control groups, indicating the effectiveness of nerve stimulation in weight control and obesity treatment. On the basis of frog leg stimulation, Lee et al. took one step forward and investigated the feasibility of using TENG for sciatic nerve and common peroneal nerve stimulation in a rat model (Figure 6c) [107]. The adopted TENG has a stacked-layer structure, with five contact-separation TENGs (micro-pyramid PDMS and Cu as the triboelectric layers) connected in parallel to enhance the output performance. After optimizing the TENG’s confinement height, it can generate a peak-to-peak voltage of 160 V and a current of 6.7 μA. The outputs are then directly applied for nerve stimulation through a flexible sling electrode as the designed neural interface. The experimental results show that direct stimulation of the rat’s sciatic nerve and the common peroneal nerve is achieved, as proven by the corresponding EMG signals being successfully recorded.

To further investigate the efficacy, selectivity, and controllability of direct stimulation on peripheral nerves, Lee et al. developed a water–air TENG array with optimized force sensitivity and sensing range, as shown in Figure 6d [170]. The suspended thin PDMS in the middle can greatly improve the generated outputs of the liquid involved TENG under pressure, i.e., from the mV range to tens of volts. Meanwhile, the adopted water-filled sponge enables a much higher sensing range compared to the hydrogel. Using the multiple pixels in the TENG array and flexible neural clip interfaces, selective stimulation of plantar flexion (PF) and ankle dorsiflexor (DF) via tibial and common peroneal nerve branches are realized on rats, with controllable muscle activation by the pressing force. The exponentially decreasing waveform from TENGs is also compared with the traditionally used biphasic square waveform, demonstrating the high effectiveness of using TENG for direct nerve stimulation. In addition to muscle control, nerve stimulation can also be used for controlling bladder function. Lee et al. from the same group investigated the direct stimulation of the autonomic pelvic nerve by a stacked-layer TENG and a flexible neural clip interface for bladder function control (Figure 6e) [171]. The study of stimulation dependence on frequency and number of pulses reveals that only two pulses (~29.5 nC per pulse) of 0.83 Hz from the TENG are sufficient to induce the micturition response in rats. These in vivo results are also compared with the stimulation results by a commercial stimulator, showing once again that the direct stimulation waveforms from TENG are more effective than the biphasic pulses.

### 5.2. Muscle Stimulation

Muscle function loss exhibits the symptoms of abnormal muscle movement and/or, more seriously, paralysis. In terms of treatment, electrical muscle stimulation to restore muscle functions is feasible as a therapeutic method. Compared to nerves that are normally concentrated in a small nerve bundle, muscles contain numerous excitable motoneurons distributed sparsely within the muscle tissues. Thus, the threshold current for muscle stimulation is much higher than that for nerve stimulation. In this regard, to achieve direct muscle stimulation from TENGs, more effective neural interface designs or higher output-performance TENGs are required, which then have been investigated by researchers [173,174,175,176].

**Figure 7:**
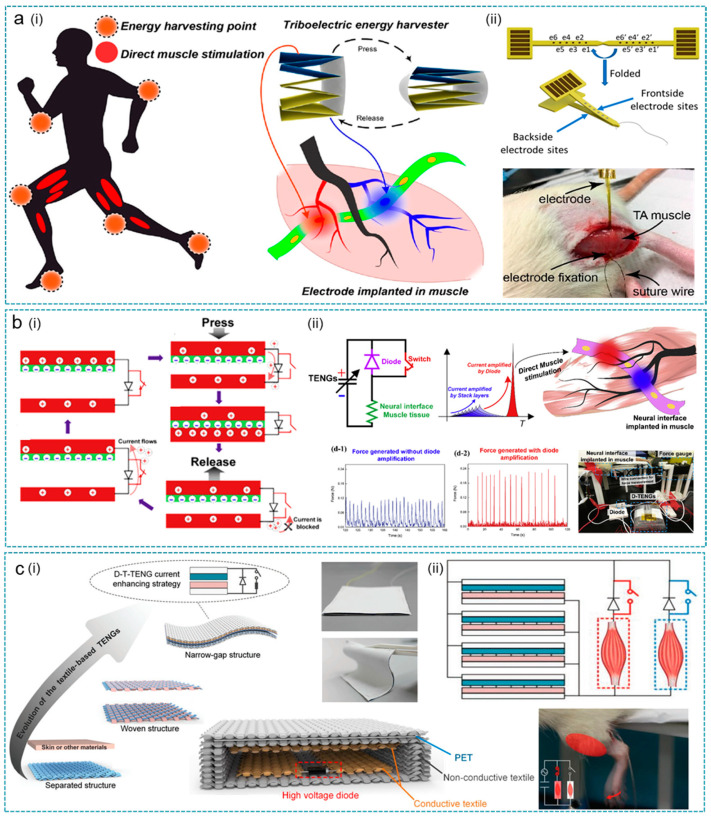
TENGs for muscle stimulation. (**a**) Direct muscle stimulation using a stacked-layer TENG and a multi-channel intramuscular electrode. The working principle (i) and in vivo experiment (ii) of the TENG using directly for muscle stimulation. Reprinted with permission from Ref. [177]. Copyright 2019 American Chemical Society. (**b**) Muscle stimulation method using a switching operation TENG to achieve higher efficiency. (i) Device working principle. (ii) The current amplified principle during muscle stimulation. Reprinted with permission from Ref. [108]. Copyright 2019 Elsevier. (**c**) A soft fabric-based thin-film TENG for selective and controllable muscle stimulation. (i) The fabrication process and (ii) working principle of the thin-film TENG. Reprinted from Ref. [178]. Copyright 2019 John Wiley & Sons.

To realize a more controllable and effective electric field in muscle stimulation, Wang et al. developed a flexible multi-channel intramuscular electrode powered by a staked-layer TENG (Figure 7a) [177]. The 12 channels (6 channels from each side) of the intramuscular electrode allow the mapping of stimulation efficiency with different channel combinations, from which a more effective stimulation can be determined even with a relatively low short-circuit current of 35 μA from the TENG. Based on the in vivo experiment on rats, it is concluded that the efficiency of electrical muscle stimulation is mainly affected by the applied waveform polarity and the electrode–motoneuron position. Therefore, when implanted in muscle, the multi-channel electrode offers great tunability in the stimulation polarity and position by connecting the outputs from the TENG to different channels, leading to a more effective direct muscle stimulation.

Along with the neural interface optimization, the performance-enhancing strategy of TENGs has also been proposed to improve muscle stimulation efficiency. Wang et al. reported a new TENG configuration, called diode-amplified TENG (D-TENG), with enhanced TENG outputs for direct muscle stimulation using a similar multi-channel neural interface (Figure 7b) [108]. As indicated, the D-TENG integrates a diode and a switch on the original stacked-TENG; thus, charges can be released instantaneously, generating a higher output current with uni-polarity (~40 μA). In vivo demonstrations show that the more efficient muscle stimulation by the D-TENG can be attributed to three aspects: (1) improvement in the current amplitude; (2) exponential waveform of the current pulse; (3) frequency increment with a shorter pulse that matches the resonance frequency of motoneurons (~500 Hz). Hence, the authors concluded that the D-TENG design is one of the most optimal configurations of TENGs for direct muscle stimulations. 

To directly use the harvested energy from body motions for muscle stimulation, wearable TENG devices based on thin and soft textiles are more convenient to be worn on various body parts. In this regard, He et al. developed a narrow-gap diode-enhanced textile-based TENG (D-T-TENG) coupled with textile switches for enhanced output currents (>100 μA), as shown in Figure 7c [178]. The D-T-TENG is fabricated using conductive textile as the electrode and Ecoflex/nitrile as the negative/positive triboelectric materials. The enhanced output currents from the D-T-TENG are then applied for direct stimulation of the anterior tibialis muscle and gastrocnemius muscle through stainless-steel wire electrodes, corresponding to leg movements of forward kicking and backward kicking a rat model. The kicking direction and force can be controlled by selecting the switches and controlling the activation area of the D-T-TENG, indicating its great applications in future prosthesis control. 

### 5.3. Cardiac Pacemaker

Artificial pacemakers provide electrical impulses to regulate the heartbeat, playing a significant role in the treatment of sick sinus syndrome, atrioventricular block, and some bradycardia-type conditions. Due to the limited life span, the battery of the artificial pacemaker needs to be surgically removed and replaced every 3–5 years, which brings great pain and surgical risks to patients. 

**Figure 8:**
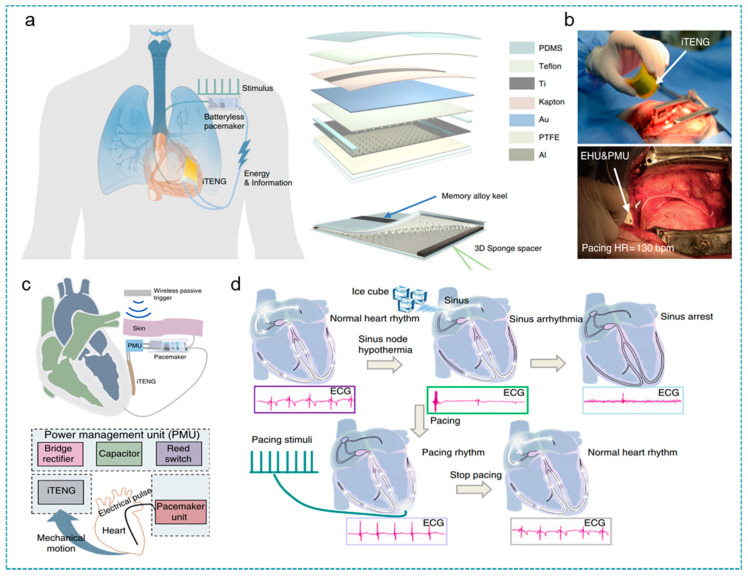
An implantable TENG for a symbiotic pacemaker. (**a**) Illustration of the symbiotic cardiac pacemaker system and structure diagram of the iTENG. (**b**) The implantation pictures of iTENG in a big animal experiment. (**c**) Illustration of a symbiotic cardiac pacemaker system in vivo. (**d**) The symbiotic cardiac pacemaker system is used to correct arrhythmia events. Reprinted from Ref. [179]. Copyright 2019 Springer Nature.

In 2014, Zheng et al. developed an implantable triboelectric nanogenerator (iTENG) in a living rat to harvest energy from periodic breathing and to be used as the direct power source in powering a prototype pacemaker for the first time. After that, as shown in Figure 8, Ouyang et al. further designed a fully implanted symbiotic pacemaker (SPM), which made progress in terms of optimizing output performance of the energy harvesting unit (iTENG), integrating the power management unit (PMU) and the pacemaker unit all in one system [179]. The iTENG was designed in a core-shell multilayer structure, with hydrophobic PDMS serving as the encapsulation and memory alloy used as the support layer. The iTENG achieved high biomechanical–electrical conversion efficiency with the electrical output of 97.5 V (V_oc_), 49.1 nC (Q_sc_), and 10.1 μA (I_sc_) in vivo, as well as the superior stability after 10^9^ times in liquid, moist (humidity of 45–50%), and dry environments. In a big animal model, driven by the heartbeat, the iTENG harvested 0.495 μJ during one cardiac cycle, which is higher than the energy required for one heart pacing (0.262 μJ).

For the cardiac pacing in a large animal model, the PMU managed the charging and peacemaking process through a wireless triggered switch. Electrical energy from iTENG was stored in a 100 μF commercial capacitance, powering the peacemaking unit to produce a 3 V, 0.5 ms electrical pulse in regulating heart rate after a wireless controller outside the body activated the control switch. Furthermore, the PMU validated the diagnosis and correction of sinus arrhythmia events in an adult Yorkshire pig. The authors created a sinus arrhythmia model using ice cubes to create sinus node hypothermia. When the abnormal ECG is detected, the pacing module starts to work, and the sinus arrhythmia is converted into a pacing rhythm. After about one minute, the SMP stopped working, and the pacing rhythm snapped back to a normal rhythm. 

The above research progress has demonstrated the TENGs as the direct electrical stimulation source for muscle function recovery, weight control, and cardiac pacing. Further neuromodulation applications, such as deep brain stimulation, vagus nerve stimulation and human functional electrical stimulation, still remain challenges. On the one hand, the frequency of the current pulses (no more than 2 Hz) generated by TENGs could not reach the requirement for neuromodulation applications, which is usually higher than 10 Hz [168]. On the other hand, direct current pulses from the TENGs need to be further amplified to a suitable magnitude for neural stimulation. Thus, the power management switches and amplifiers should be integrated into the neuro-stimulation system. 

## 6. Advanced IMDs Based on Functional TENGs

### 6.1. Biodegradable and Absorbable TENGs

**Figure 9:**
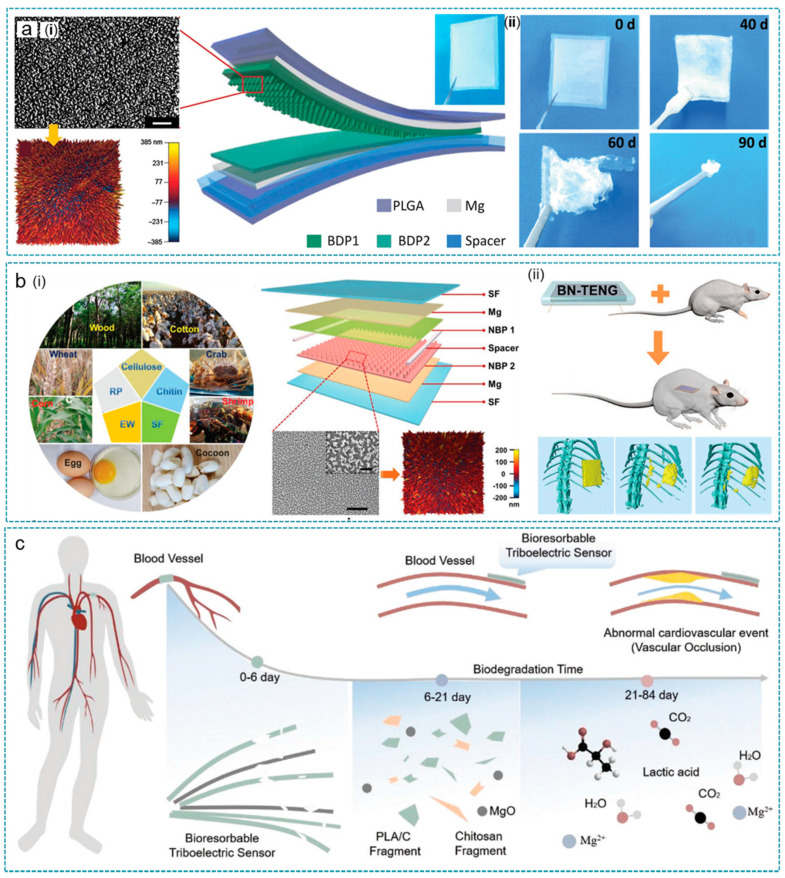
Degradable and absorbable TENGs. (**a**) Biodegradable TENG (BD-TENG) as a lifetime designed implantable power source. The structure diagram (i) and in vitro degradation process (ii) of the BD-TENG. Reprinted from Ref. [180]. Copyright 2016 American Association for the Advancement of Science. (**b**) The structure of fully bioabsorbable natural-materials-based TENGs (i) and the in vivo biodegradation process (ii). Reprinted with permission from Ref. [189]. Copyright 2020 American Chemical Society. (**c**) A bioresorbable dynamic pressure sensor based on TENG. Reprinted with permission from Ref. [93]. Copyright 2021 John Wiley & Sons.

IMDs play significant roles in patient care and disease management [49,180,181,182,183]. However, after their service, almost all implantable electronic devices need to be removed or replaced by invasive, complicated surgery [184,185,186,187]. IMDs that are degradable and absorbable in the body provide a feasible solution to avoid secondary invasive procedures [23,188,189,190]. In 2016, Zheng et al. reported a biodegradable triboelectric nanogenerator (BD-TENG) for in vivo energy harvesting with the fully bio-degradable polymers (BDPs) as the friction and encapsulation layer, the resorbable Mg as electrodes (Figure 9a) [180]. A series of TENGs based on several pairs of BDPs as friction layers compared BDP’s relative ability to gain or lose electrodes during the triboelectric process, which achieves a voltage output ranging from 20–40 V and different degradable rates at the same time. In an adult SD rat model, the PLGA-encapsulated BD-TENG output voltage decreases from 4 V to 1 V within 2 weeks, failed in about 4 weeks, and completely lost its structure after 9 weeks, indicating the feasibility of degradable in vivo. After that, in the same group, Jiang et al. further developed fully bioabsorbable natural-materials-based triboelectric (BN-TENG) nanogenerators based on cellulose, chitin, silk fibroin (SF), rice paper (RP), and egg white (EW) (Figure 9b) [189]. The authors verified the triboelectric series of these five materials as EW > SF > Chitin > Cellulose > RP, which provides a reference for future BN-TENGs in materials selection and structure design. With different pairwise combinations, the as-fabricated BN-TENG realized a large range of output performance with V_oc_ ranging from 8 to 55 V and I_sc_ ranging from 0.08 to 0.6 µA. The degradation rate of BN-TENG can be regulated by adjusting the fibroin crystallinity with methanol, achieving device failure time in vivo ranging from 24 h to 11 d, and then complete degradation within 84 days. 

In addition, active sensors with biodegradable and absorbable properties also made a breakthrough. As shown in Figure 9c, Ouyang et al. designed a bioresorbable dynamic pressure sensor based on the triboelectric effect (BTS), which can convert biological pressure signals into electrical signals [93]. The BTS has excellent sensing characteristics, achieving sensitivity up to 11 mV/mmHg and good linearity of R^2^ = 99.3%. In the large animal model (dog), BTS achieved accurate monitoring of pleural pressure, vascular pressure, and identification of vascular occlusion events. In addition, the poly(lactic acid)-(chitosan 4%), which serves as both friction and adhesion layer, equipped the BTS outstanding antibacterial ability of 99% sterilization, making the implantable BTS free from the risk of tissue infection. The implanted BTS can be completely degraded after its service and completely be absorbed within 21–84 days. 

The development of implanted TENGs based on degradable materials has strongly promoted the progress of next-generation customizable IMDs [191,192]. Since degradable materials were first applied to TENGs, more than a dozen degradable materials have been developed [193,194]. They have been applied in energy harvesting, mechanical sensing, and even implantable electrical stimulation [195,196]. In the future, the design of degradable materials and the performance optimization of degradable TENGs are still full of challenges, and more research and exploration are needed.

### 6.2. Self-Healing TENGs

The performance of TENGs relies on the dynamical process of converting bio-mechanical energy into electrical energy [197,198]. At the same time, the long-term repeat mechanical deformation also led to different mechanical and functional damages of TENG: including cracks, mechanical fatigue, degradation of friction surface structure [199,200]. Developing advanced material with self-healing ability helps damaged TENGs recover their mechanical morphology and functions, which contributes to prolonging their service life and usage safety [201,202,203].

**Figure 10:**
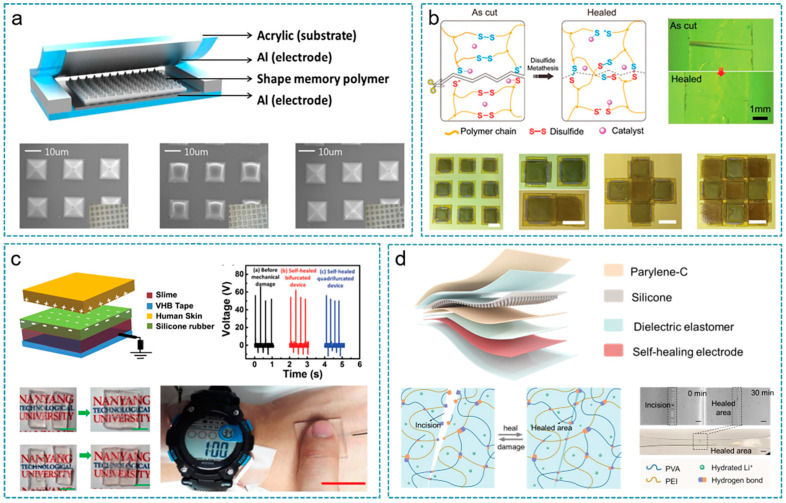
Self-healing TENGs. (**a**) Shape memory polymer-based self-healing TENG. Reprinted with permission from Ref. [204]. Copyright 2013 Royal Society of Chemistry. (**b**) Vitrimer elastomer-based jigsaw puzzle-like healable TENG for self-powered wearable electronics. Reprinted with permission from Ref. [200]. Copyright 2018 John Wiley & Sons. (**c**) Highly transparent, stretchable, and self-healing ionic skin for energy harvesting and touch applications. Reprinted with permission from Ref. [201]. Copyright 2017 John Wiley & Sons. (**d**) Structure of the stretchable, self-healing, and skin-mounted muscle sensor for muscle function assessment. Reprinted with permission from Ref. [205]. Copyright 2021 American Chemical Society.

In 2013, Lee et al. demonstrated the first self-healing TENG based on a shape memory polyurethane (SMPU) triboelectric layer, with an original output of 80 V and 3 μA, respectively (Figure 10a) [204]. A series of compression forces were applied on the SMPU-TENG to investigate the degradation of the micropatterned triboelectric layer. With a high compression force of 10 kgf for 15 min, the performance of SMPU-TENG reduced by about 90%. By raising the temperature to T_g_ = 55 °C to trigger the healing process, the SMPU micro-pyramid pattern recovers its initial shape and performance to its original state. 

Deng et al. then developed a self-healing vitrimer elastomer and successfully used it in the single-electrode mode TENG (Figure 10b) [200]. Based on the break and reform of dynamic disulfide bonds, the vitrimer elastomer realized 100% self-healing efficiency after extreme damage when exposed to 90 °C high temperatures within 20 min and intense pulsed light within the 30 s. Embedded with silver wires, the elastomer demonstrated superior conductivity and was utilized as a matrix to construct TENG, which performs outstanding output with I_sc_ reaching 350 nA and V_oc_ reaching 26 V. The self-healing ability of the vitrimer elastomer not only helps TENG recover from accidental injury but also provides a simple method to assemble TENGs into desired 2D and 3D structure free of glue with the mechanism of jigsaw puzzles and building blocks toys. At the same time, the VTENGs in 3D structure increased to about 450% charging efficiency as the unit increased from one to nine and showed satisfactory sensitivity to external motion when used as sensing elements and wearable electronics. 

Compared with other components, the electrode of flexible TENGs is more vulnerable to damage, for they are mostly based on a thin layer of metal deposition or conductive composite. In 2017, Parida et al. reported a highly transparent, stretchable, and self-healing TENG (IS-TENG) for energy harvesting and touch application, using a slime-based ionic conductor as the self-healing electrode for the first time (Figure 10c) [201]. The IS-TENG based on the ionic conductor as current collector achieved a charge collecting efficiency 12 times higher than that based on silver, which exhibited 92% transparency and over 700% stretchability at the same time. The IS-TENG behaved with excellent self-healing ability and recovered its performance in energy harvesting and powering small wearable electronic devices after 300 times complete bifurcation and self-healing at room temperature. 

Furthermore, Wang et al. designed an ultra-stretchable and fast self-healing ionic hydrogel based on PVA/PEI/LiCl (Figure 10d), which acts as a current collector in the active muscle sensor based on a single electrode mode TENG (TSAS) [205]. The ionic hydrogel exhibited superior self-healing ability at room temperature, achieved complete recovery within 30 min, and further afforded more than 2000% tensile deformation without breaking. At the same time, the conductivity recovered 98.57% within 4 min after bringing damaged samples together and maintained over 96% healing efficiency after 10 times extreme damage. The as-fabricated TSAS performed outstanding sensing properties with a detection limit of 0.2 mN, a response time of 1 ms, and cycle stability of up to 10^5^. In the in vitro experiments, the authors set up a quantitative relationship between muscle force and output of TSAS, and they developed a real-time muscle function monitoring system through combining TSAS with wireless transmission and visualization terminal.

Self-healing TENG plays an important role in improving the service life and safety of the IMDs [206,207,208,209]. The 2D and 3D modular assembly of the IMDs, with the help of self-healing ability, greatly simplifies the tedious fabrication process [210,211]. However, the current self-healing TENGs mainly focus on wearable electronic devices and remain blank for implantation scenarios [212,213,214,215]. The self-healing efficiency in liquid environments, compatibility with other components, and the development of fully self-healing systems are major challenges affecting in vivo applications [216,217,218]. Therefore, optimizing the self-healing performance in vivo and rationally designing the self-healing devices’ structure are feasible solutions for the expanding application scenarios of self-healing TENGs in vivo.

## 7. Discussion and Challenges 

Despite implantable TENGs having made significant progress in self-powered sensors, in vivo energy harvesters, and direct electrical stimulators, aided by improved materials, structural design, performance, and applications, some unsolved challenges still require continuous attention. Here, we discuss the optimization directions of IMDs and their prospects mainly from three aspects of performance, advanced functional materials and the close-looped IMDs system. 

### 7.1. Output Performance

**Figure 11:**
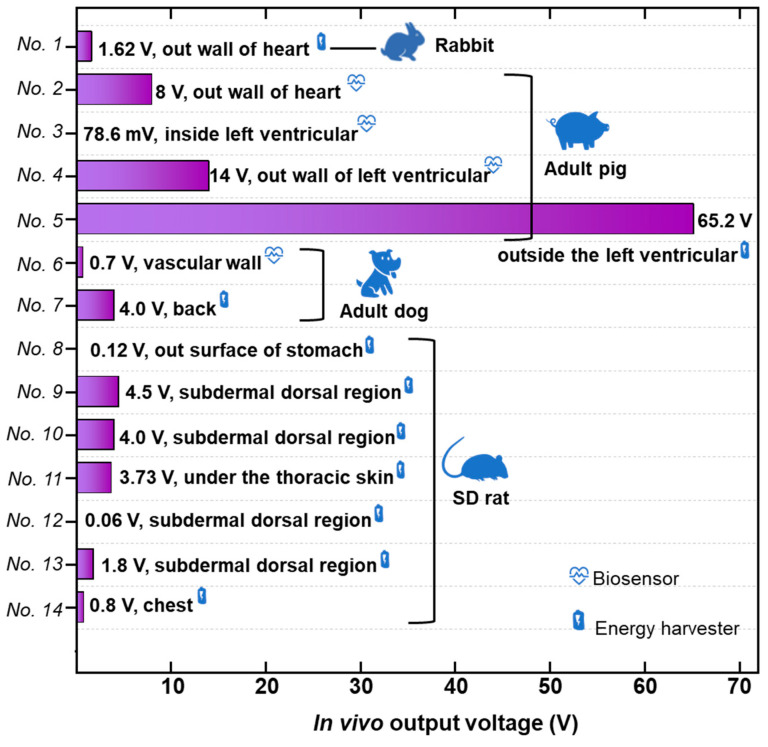
The in vivo output voltage of IMDs. Ref: No. 1-Shi et al., 2019 [92]. No. 2-Ma et al. 2016 [102]. No. 3-Liu et al. 2019 [103]. No. 4-Zheng et al. 2016 [3]. No. 5-Ouyang et al. 2019 [180]. No. 6-Ouyang et al. 2021 [93]. No. 7-Ryu et al. 2021 [5]. No. 8-Yao et al. 2018 [109]. No. 9-Jiang et al. 2018 [190]. No. 10-Zheng et al. 2016 [181]. No. 11-Zheng et al. 2014 [144]. No. 12-He et al. 2019 [58]. No. 13-Li et al. 2018 [191]. No. 14-Li et al. 2018 [105].

Higher output performance is a key factor for IMDs. We summarized the output of TENG-based implanted energy harvesting devices [102,103,104,219,220,221,222]. As shown in Figure 11, the electrical output is not only related to the performance of the device but also affected by the implanted animal, and implantation site. To increase the output, a variety of methods were proved to be effective, increasing the surface charge density as well as the contact area of two friction materials. Two frictional materials with a large difference in triboelectric sequence generate more triboelectric charges and transfer charges more efficiently [223,224,225]. The surface charge density could be increased by injecting charges into the friction layers using the corona discharge method [179]. Increasing the relative contact area between two friction layers proved to be another effective method, such as grinding the fractional surface by sandpaper, ICP to form pillar-shaped, pyramid-shaped, and origin-shaped surface structures [226]. The connection of multiple devices is considered a common and effective method to increase output [144]. In addition to the above-mentioned, matching external loads and effective energy management modules should also be considered for better output performance. Furthermore, considering the liquid environments in the human body, the electrostatic shielding effect should be reduced during the design of the IMDs.

Another important point is the implantation site. For cardiac function monitoring, the IMD output varied when implanted on the right ventricular, the auricle of the left atrium, the cardiac base, the lateral wall of the left ventricular, and the inferior wall of the left ventricular, for different motion amplitude and volume of the interspace of these implantation sites. Therefore, a reasonable implantation site is suggested to optimize the output of IMDs and reduce the organism damage as much as possible.

### 7.2. Advanced Materials with Multifunctions

Another significant issue is the safety, reliability, and high efficiency of IMDs. Significances should be taken into consideration for the large differences among the different application scenarios [48,56,227,228]. In Table 2, we summarized the IMDs based on advanced TENG with various friction materials and made a comparison about their performance and applications. For IMDs working in the stomach, acid-resistant encapsulation is required to resist gastric acid erosion and better tissue-matched modulus because the stomach is constantly peristaltic [109]. Biosensors used for blood pressure monitoring must have anti-hemolytic properties to reduce the impact on the monitoring site [103]. Meanwhile, almost IMDs are generally subject to mechanical losses and accidental damage during operation, resulting in mechanical fatigue and material wear of the device, thereby affecting its service life. Others need surgical removal after service, which increases patient suffering and the risk of infection. Developing functional materials with better mechanical performance, biodegradable and self-healing ability, antibacterial ability, may provide promising solutions.

### 7.3. Close-Looped IMDs System

A complete close-looped IMDs system that starts with accurate real-time physiological information collection and gives corresponding feedback and guidance is growing as the main development demand of modern intelligent electronic medicine [227]. Various health monitoring devices and treatment tools emerge in an endless stream but mainly work independently as active biosensors, power sources, and other functional units [22]. On the other hand, the development of self-powered technology has made IMDs break through the limitation of service life and realize long-term real-time monitoring/treatment of the human body [28]. With the gradually reduced power consumption of electronic devices and the continued development of advanced integration technologies, it is possible to develop a self-driven close-looped bioelectronic system with the human body as the energy source to power the continuous working of the whole system [48,78]. 

In a close-looped IMDs system, the energy harvesting units collect the various types of bioenergy from the human body and convert them into electrical energy, which is further rectified by the power management module and then stored in the power storage unit or directly supplied to other energy-consuming units. Without additional energy supply, the self-powered sensors perceive the real-time physiological states of the human body and deliver the sensing dates to the processing unit through a wireless transmission module. Once the dates are diagnosed as anomalous, instructions from the controller will be sent to the relevant downstream functional units so as to perform feedback adjustment on the corresponding tissues, organs, or nerves of the human body until the physiological states are back to normal. With the help of the close-looped IMDs system, the physiological state of the human body can be automatically adjusted to the optimal state, and when a disease occurs, the physiological system can be actively and intelligently repaired.

## 8. Conclusions

Biomedical systems and advanced nanotechnology are sparking a new revolution in the field of healthcare. The continuous reduced power consumption of microchips and the continuously improved working efficiency of micro–nano energy harvesting devices have made fully self-charged implantable biomedical devices (IMDs) possible. Freeing the IMDs from the bulk batteries is a key step during this process. In this review, we summarized the recent progress of advanced IMDs driven by TENGs and reviewed the research progress of self-powered biosensors, bioenergy harvesting devices, and electrical stimulation therapy devices. For the self-charged IMDs, it is important to improve the output performance of the implantable TENGs and optimize the energy management module. The flexible packaging technology with high stability and high biosafety is required to ensure high performance in liquid environments. Besides, the good adaptability of these IMDs to the shape of human tissues or organs will improve sensor performance and energy harvesting efficiency.

The rapid development of advanced electronic materials and fabrication technology has ushered in a new opportunity to develop a fully self-charged IMD system. The integration of multi-functional components of the whole system is currently the main challenge. Advanced self-charged IMDs with self-healing properties enable a longer lifetime in devices in accidental damage, and also help achieve 3D expansion of multiple functional devices, which breaks the fabrication process limitations of traditional integrated systems [205,229]. At the same time, the proposal and development of the body net provides another distributed integrating method [157,230,231,232,233]. Each functional unit communicates through a local wireless network to realize a close-looped system integrating in suit sensing, data analysis, and treatment, which will make the implantable electronic medical system more intelligent. 

## Figures and Tables

**Table 1:** The summary of the self-powered biosensors.

Implant Site	Animal Model	Output In Vitro	Size	Application	Implantation Time	Ref.
Heart (out wall)	Adult pig	14 V, 5 µW	2.5 × 1 × 0.15 cm^3^	Heart rate,	72 h	[3]
Heart (out wall)	Adult pig	10 V, 4 µA	3 × 2 × 0.1 cm^3^	Heart/respiratory rate, atrial fibrillation, ventricular premature contraction	2 weeks	[102]
Heart (LV) ^a^	Adult pig	6.2 V	10 × 5 × 1 mm^3^	Endocardial pressure	—	[103]
Bladder	Adult rat	114 mV	1.4 × 2.2 cm^2^	Bladder pressure	—	[104]

^a^ LV: left ventricular.

**Table 2:** Performance comparison of TENGs with different triboelectric materials.

Property	Friction Materials	Electrodes	Optimal Output	Size	Application	Ref.
Robust	PTFE/caption	Cu/Al	70 V, 0.8 μA, 10.5 nC	6 × 4 × 0.15 cm^3^	Energy harvesting	[188]
Implantable	PTFE	Cu/Ti	70 V, 0.55 µA, 25 nC	—	In vivo cancer therapy	[184]
	PFA	Au/Cu	9.71 V, 427 µA	4 × 4 cm^2^	Ultrasound driven energy harvesting	[106]
	PTFE	Cr/Cu	2.2 V, 0.1 µW	—	Breath energy harvesting	[105]
	PTFE	Cu/Al	14 V, 5 µW	2.5 × 1 × 0.15 cm^3^	Cardiac monitoring	[3]
	caption	Cu/Al	22 V, 0.3 µA	—	Energy harvesting	[51]
	silicone	Au	16.8 mV, 54.4 nW	—	Ultrasound driven energy harvester	[155]
	PTFE	Au/Al	6.2 V	10 × 5 × 1 mm^3^	Endocardial pressure sensor	[103]
	caption	Au/Al	12 V, 0.25 µA	1.2 × 1.2 cm^2^	Powering prototype pacemaker	[144]
	caption	Au/Al	187 V, 19.5 μA,.80.2 nC	3.9 × 6.1 × 0.099 cm^3^	Powering cardiac pacemaker	[179]
	^a^ PVA-NH_2_/PFA	Au/Cu	136 V, 2 μA/cm^3^	15 mm in radius, 2.4 mm in height	Powering cardiac pacemaker	[5]
^b^ BA-TENG	^c^ cellulose/chitin/SF/RP/EW	Mg	55 V, 0.6 µA, 12 nC	—	Energy harvesting	[189]
	^d^ PLA/C	Mg	3.2 V	—	Blood pressure sensor	[93]
	^e^ PLGA/PCL/PLA	Au	28 V, 220 nA	1.2 × 1.2 cm^2^	Tissue repair	[190]
^f^ SH-TENG	SH-PCL/PVDF	Cu/AgNWs	800 V, 28 μA	3 × 3 cm^2^	Energy harvesting	[197]
	Parylene-C/silicone	^f^ SH-IH	78.44 V, 1.42 μA, 47.48 nC	3 × 5 cm^2^	Muscle strength sensor	[205]
	vitrimer elastomer	Al/AgNWs	26 V, 350 nA, 10 nC	10 × 3 cm^2^	Energy harvesting, pressure/tactile senser	[200]
	silicone	slime	50 V, 6.5 µA/cm^2^, 17 nC/cm^2^	2 × 2 cm^2^	Energy harvesting	[201]
	shape memory polymer	Al	100 V, 15 µA	4 × 2.5 cm^2^	Energy harvesting	[204]
	SH-PUA/latex	^g^ PUA-SF-LM	100 V, 4 μA/cm^2^, 12 nC/cm^2^	3 × 3 cm^2^	Energy harvesting	[209]

^a^ PVA-NH2: Amine-functionalized poly (vinyl alcohol); PFA: Perfluoroalkoxy. ^b^ BA: biodegradable and absorbable. ^c^ SF: silk fibroin; RP: rice paper; EW: egg white. ^d^ PLA/C: poly (lactic acid)–(chitosan 4%); ^e^ PLGA: poly (L-lactide-co-glycolide); PCL: poly (caprolactone). ^f^ SH: Self-healing; IH: ionic hydrogel. ^g^ SF: silver flakes; LM: liquid metal.

## Data Availability

No new data were created or analyzed in this study. Data sharing is not applicable to this article.
